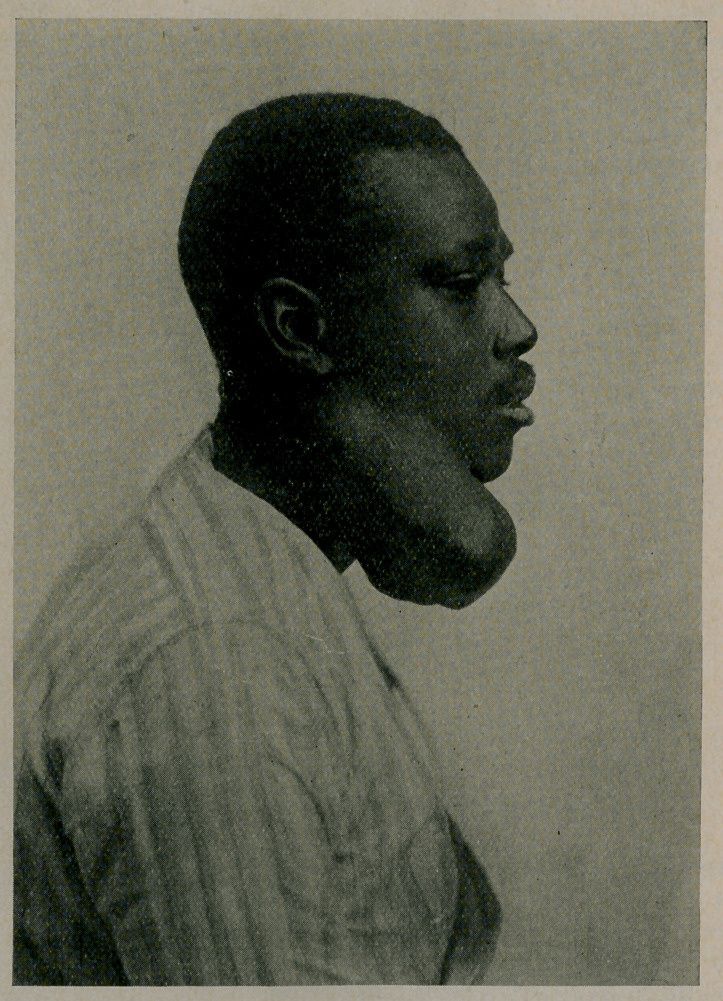# Solid Tumors of the Sub-Maxillary Gland

**Published:** 1914-01

**Authors:** 


Solid Tumors of the Sub-maxillary Gland. Addison G. Brenizier, A. B., M. D., Charlotte, N. C., *Old Dominion Jour. of Med. & Surg.,* Nov., 1913. The author reports a case in a negro aged 22, of a typic mixed tumor which had grown from the size of a walnut in seven years. (Cuts reproduced by courtesy of editor.) He also alludes to two cases of malignant recurrence after incomplete operation, one in a physician's wife, aged 34, the other in a man of 24.

Tumors of the sub-maxillary gland are relatively rare. Kuettner, in 1897, was able to gather together ninety-seven cases scattered in the literature, to which he added six other unrecorded cases. Since this date a few more cases have been reported by different authors.

Among 12,850 surgical cases at the Johns Hopkins Hospital, Bloodgood found but five tumors of sub-maxillary gland. (Chronic inflammations are excluded from this number.)

All statistics show that tumors of the sub-maxillary gland are much rarer than those of the parotid gland—a proportion of about one to nine. As with the parotid, a great majority of tumors of the sub-maxillary are the so-called mixed tumors, and these tumors present the same gross and microscopical picture in both glands. Out of seventy-eight cases where the histologic picture was shown, Kuettner reports sixty-five mixed tumors. (Kuettner indicates fifty-eight mixed tumors and seven endotheliomata, but the distinction between the two varieties is open to discussion.) The other varieties of tumors of the sub-maxillary gland are indeed rare. Up to 1900 there were only four cases of a pure sarcoma observed (Jouliard, Volkmann, Lotheissen and Bloodgood), and three cases of adenoma (Talazac, Duplay and Poncet). As to carcinoma, Kuettner cites five cases, of which only two appear certain (Waldeyer and Volkmann).

Clinically, Kuettner distinguishes two groups of tumors of the sub-maxillary gland: tumors showing a benign evolution, embracing mixed tumors and adenomata, and tumors showing a malignant evolution, the sarcomata and the carcinomata.

## Figures and Tables

**Figure f1:**
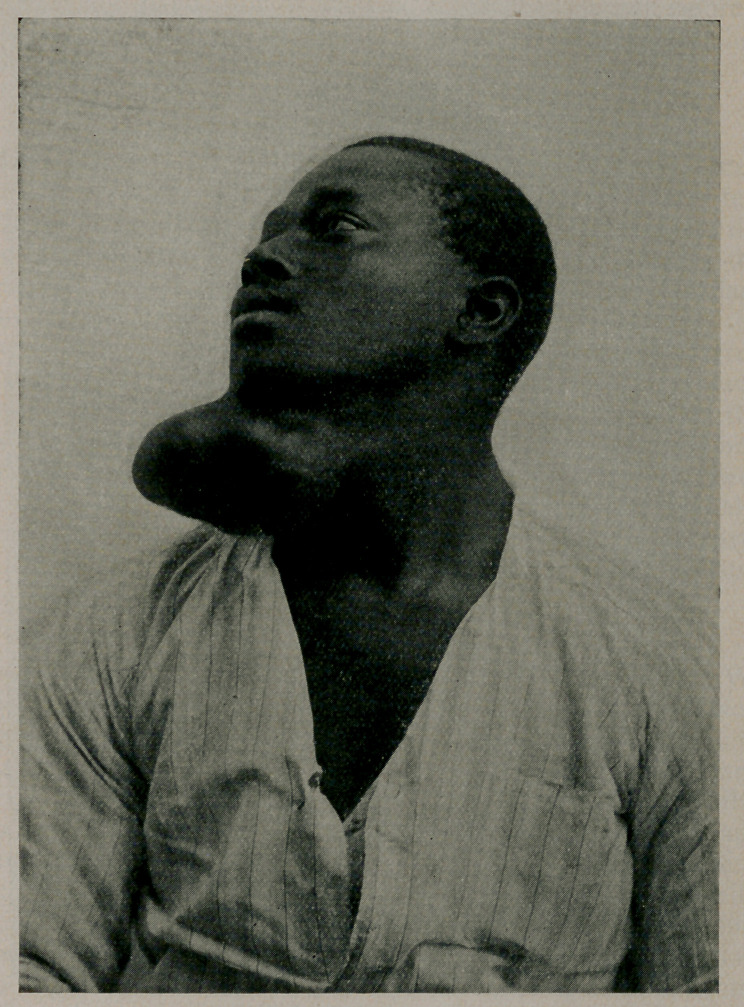


**Figure f2:**